# Protein-Stabilized Palm-Oil-in-Water Emulsification Using Microchannel Array Devices under Controlled Temperature

**DOI:** 10.3390/molecules25204805

**Published:** 2020-10-19

**Authors:** Takashi Kuroiwa, Miki Ito, Yaeko Okuyama, Kanna Yamashita, Akihiko Kanazawa

**Affiliations:** 1 Department of Chemistry and Energy Engineering, Faculty of Science and Engineering, Tokyo City University, 1-28-1 Tamazutsumi, Setagaya, Tokyo 158-8557, Japan; g1781901@tcu.ac.jp (M.I.); g1881219@tcu.ac.jp (Y.O.); g1516066@tcu.ac.jp (K.Y.); akanaza@tcu.ac.jp (A.K.); 2 Advanced Research Laboratories, Tokyo City University, 8-15-1 Todoroki, Setagaya, Tokyo 158-0082, Japan

**Keywords:** lipid microsphere, solid/semi-solid lipids, microchannel emulsification, monodisperse emulsion, multiple emulsions, sodium caseinate

## Abstract

Microchannel (MC) emulsification for the preparation of monodisperse oil-in-water (O/W) and water-in-oil-in-water (W/O/W) emulsions containing palm oil as the oil phase was investigated for application as basic material solid/semi-solid lipid microspheres for delivery carriers of nutrients and drugs. Emulsification was characterized by direct observation of droplet generation under various operation conditions, as such, the effects of type and concentration of emulsifiers, emulsification temperature, MC structure, and flow rate of to-be-dispersed phase on droplet generation via MC were investigated. Sodium caseinate (SC) was confirmed as the most suitable emulsifier among the examined emulsifiers, and monodisperse O/W and W/O/W emulsions stabilized by it were successfully obtained with 20 to 40 µm mean diameter (*d*_m_) using different types of MCs.

## 1. Introduction

Solid/semi-solid lipid microspheres are utilized for powderized (encapsulated) fats including coating fats, as well as delivery carriers for hydrophobic bioactive compounds, and have attracted much attention in the food and pharmaceutical industries [1,2,3,4,5]. For utilization as delivery carriers, solid/semi-solid lipids have advantages over those consisting of liquid oils, as the high barrier ability of solidified matrices and their variable physicochemical properties due to phase transition enable control of the entrap/release properties of encapsulated bioactive compounds including nutrients and drugs [2,3,6,7,8,9]. Recently, multiple particles consisting of crystallizable fats that entrap fine aqueous droplets have also been studied regarding their encapsulation ability and stability due to unique barrier effects and controlled release properties of not only lipophilic but also hydrophilic compounds. However, the number of reported examples is limited [10,11,12].

For these applications, it is important to clarify the mechanisms of destabilization of oil droplets or lipid microspheres during their preparation and storage, and to control their release properties by designing colloidal systems with responsiveness to various external stimuli. However, the phase behavior of crystallizable lipids in colloidal systems, especially those consisting of natural oils and fats, is often very complicated, and their crystallization behaviors, including crystallization temperature, and orientation of lipid crystals, differ from those of bulk lipids [2,13,14,15,16,17]. In this context, studies on colloidal dispersions containing crystallizable lipids have risen in importance in the field of fundamental science as well as in various applications.

In this study, we selected palm oil as the solid/semi-solid material of lipid microspheres. Palm oil is obtained from fruits of the oil palm tree and contains mainly palmitic, oleic, and linoleic acids (>90% of their total content), with almost 50–50 composition of saturated and unsaturated fatty acids [18,19,20,21]. A solid/semi-solid vegetable fat with a melting point of 30 to 35 °C, palm oil exhibits complicated polymorphology [22]. Because of its unique properties, it is utilized not only as edible oil including cocoa butter equivalents, but also as a raw material of soap and oleochemical products. Recently, palm oil has attracted much attention because it is a naturally occurring solid fat that is free of trans fatty acids, whereas hydrogenation of liquid vegetable oils results in generation of trans fatty acids in the food industry. Furthermore, palm-oil-based formulations have recently been considered for developing effective drug delivery systems [23]. Therefore, we believe that investigation of the emulsification of palm oil is important for clarifying the preparation characteristics of solid lipid microspheres in practical fields.

Solid lipid microspheres are usually prepared from oil-in-water (O/W) emulsions stabilized by a water-soluble emulsifier at a temperature above the melting point of the dispersed lipid phase [3]. It is important to maintain the temperature of the emulsion appreciably above the crystallization temperature of the highest melting lipid component throughout emulsification to prevent solidification of the lipid phase. The prepared hot emulsion is then cooled so that some or all of the dispersed lipid droplets crystallize. The size of the solid lipid microspheres produced generally depends on the initial size of the droplets in the hot emulsion; therefore, it can be controlled by the selection of emulsification methods and conditions. Since the size of the solid lipid microspheres affects the physical properties and delivery performance of foods or pharmaceutical preparations, size control of the solid lipid microspheres and the starting hot emulsion droplets is crucial in their production.

This study focuses on the formation of palm-oil-in-water emulsions under controlled temperature using microchannel (MC) emulsification [24,25]. MC emulsification can produce highly uniform droplets by pressing a dispersed phase into a continuous phase through MCs fabricated on single crystal silicon, polymer, or stainless-steel chips. Its unique emulsification properties with an interfacial-tension-driven process have been characterized both experimentally and computationally [25,26]. Furthermore, droplet formation in MC emulsification can be observed directly and individually under a microscope; therefore, this technique is advantageous for characterizing the behavior of the liquid-liquid interface during emulsification in detail. To date, emulsion-based microspheres consisting of various materials including polymeric particles [27,28,29] and hydrogel beads [30,31,32], as well as solid lipid microspheres [33,34], have been developed using MC emulsification. Especially for the solid lipid microsphere with a high melting temperature, temperature control during emulsification is essential for obtaining hot emulsions as the precursors of microspheres. However, only a few studies using high-temperature MC emulsification have been conducted [31,35,36,37], and only one study preparing hot emulsions using solid/semi-solid lipids has been reported [33]. Thus, in the present study, MC emulsification using palm oil was carried out above its crystallization temperature with food grade emulsifiers including nonionic and protein emulsifiers, with the intention of obtaining biocompatible lipid microspheres. We also aimed to prepare multiple palm oil droplets containing small water droplets encapsulating a water-soluble molecule. The results of these experiments provide useful information about preparation characteristics of palm-oil-based microspheres and related materials from both scientific and technical viewpoints.

## 2. Results and Discussion

### 2.1. Preparation of Palm-Oil-in-Water Emulsions by MC Emulsification

#### 2.1.1. Effect of Emulsifier Type

**Figure 1:**
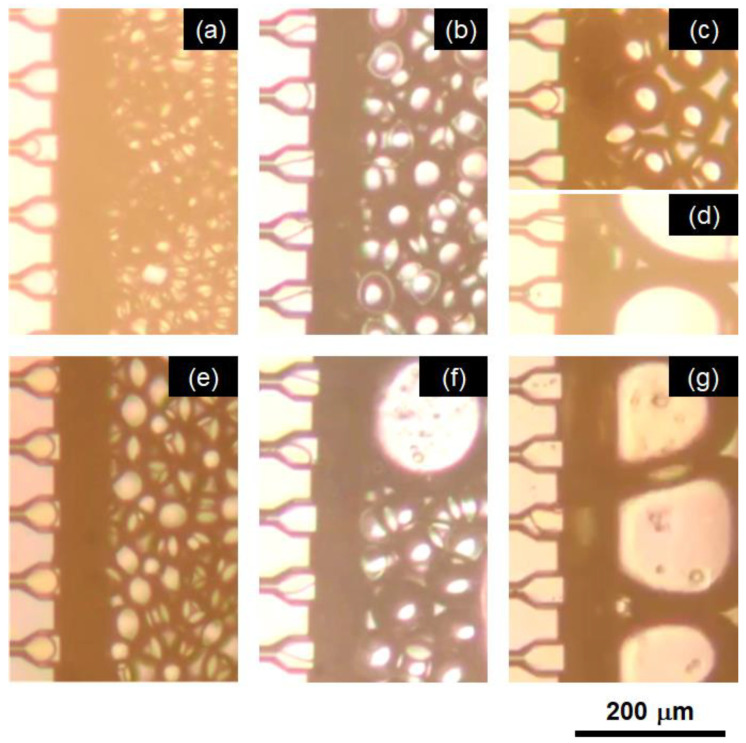
Effect of emulsifier types on emulsification at 60 °C with microchannel plate type A (MC-A, see Section 3.2.) using (**a**) sodium dodecyl sulfate (SDS), (**b**) hexaglycerin monolaulate (HGML), (**c,d**) Tween 80, (**e**) sodium caseinate (SC), (**f**) β-lactoglobulin (β-LG), and (**g**) skimmed milk powder. The concentration of each emulsifier was 3 wt%.

First, the effect of the type of emulsifier added to the continuous aqueous phase was investigated. A dead-end plate with MC-A was used, and the emulsification temperature was set to 60 °C (see “Materials and Methods” for details of the MC emulsification setup). One anionic (sodium dodecyl sulfate: SDS), two nonionic (hexaglyceryl monolaurate: HGML, and Tween 80), and two protein emulsifiers (sodium caseinate: SC, and β-lactogloburin: β-LG), as well as a food-grade skimmed milk powder were dissolved in water at 3 wt% and used for the emulsification experiments. Photomicrographs of emulsification using each emulsifier are depicted in Figure 1. Uniform oil droplets formed using SDS (Figure 1a) and SC (Figure 1e) as emulsifiers. Droplet formation was also observed for nonionic emulsifiers HGML (Figure 1b) and Tween 80 (Figure 1c), although the uniformity of droplet diameter decreased due to partial wetting of the MC wall with the oil phase. For Tween 80, the oil phase intermittently flowed out at several MCs (Figure 1d). This unstable emulsification behavior might be due to a change in the hydrophilicity of the head groups of these nonionic emulsifiers at relatively high temperature. Similar wetting of MC walls and continuous expansion of the dispersed phase at the end of MCs were also observed using β-LG as an emulsifier (Figure 1f), although successful droplet formation was achieved using SC (Figure 1e), which was a component of milk proteins, as well as β-LC. With skimmed milk powder, the oil phase did not detach from the terrace part and flow out continuously (Figure 1g), even though the skimmed milk powder contained over 30% casein.

**Figure 2:**
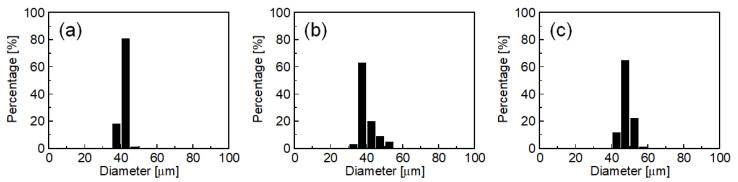
Number-weighted droplet diameter distributions of oil-in-water (O/W) emulsions obtained by MC emulsification at 60 °C with MC-A using (**a**) SDS, (**b**) HGML, and (**c**) SC as emulsifiers. Each histogram contains the data on diameter for more than 100 droplets.

Figure 2 plots droplet diameter distributions of O/W emulsions obtained using SDS, HGML, and SC with the better emulsification. For SDS, the mean diameter (*d*_m_) was 41.0 µm, and the coefficient of variation (CV) was 2.9%; for HGML, *d*_m_ was 39.6 µm, and CV was 11.4%; for SC, *d*_m_ was 47.9 µm, and CV was 5.9%. In MC emulsification, various features of emulsifiers reportedly affect the droplet formation behavior. To clarify the role of type of emulsifier, it is important to evaluate the effect of various characteristics of the emulsifier such as interfacial tension, contact angle, charge property, and hydrophilic-lipophilic balance values. However, these challenges would be addressed in our future study. Considering good emulsification ability and biocompatibility as a food feature, here we selected SC as the emulsifier for the emulsification experiments described in the following sections.

#### 2.1.2. Effect of SC Concentration

**Figure 3:**
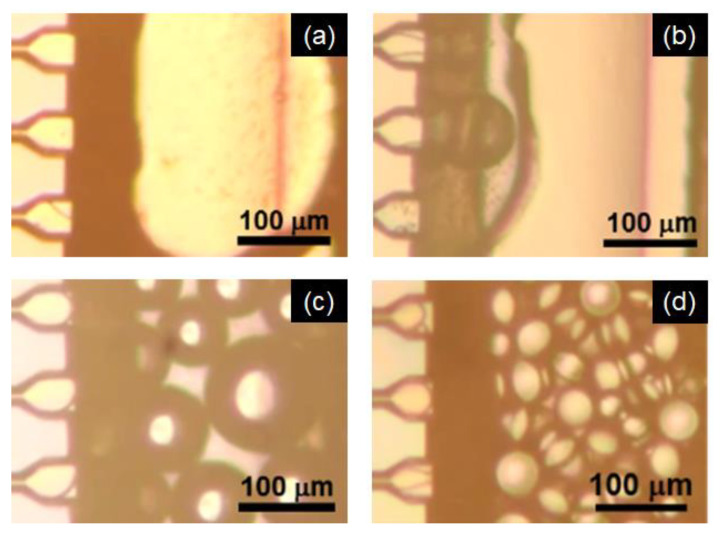
Droplet generation by microchannel (MC) emulsification at 60 °C with MC-A using (**a**) 0.1, (**b**) 0.5, (**c**) 1.0, and (**d**) 3.0 wt% SC as an emulsifier.

Figure 3 depicts emulsification at different emulsifier (SC) concentrations in the continuous aqueous phase. At 0.1 and 0.5 wt% SC concentrations (Figure 3a,b), continuous flow out of the palm oil was observed; thus, it was impossible to produce O/W emulsions with these SC concentrations. At 1 wt% (Figure 3c), oil droplets were generated at the downstream of MCs, but continuous flow out and immediate coalescence of generated droplets were observed in some MC regions. At 3 wt% SC concentration (Figure 3d), uniform oil droplets with *d*_m_ = 48 µm (CV < 6%) were obtained. These results were attributed to the interfacial activity of SC at different concentrations. According to our measurement, the values of interfacial tension at SC concentration above 0.5 wt% ranged from 8.7 to 9.0 mN/m, indicating no significant difference. As illustrated in Figure 3, however, emulsification remarkably improved at SC concentration exceeding 0.5 wt%. As reported previously [38], the emulsifier concentration at the end of MC is in a different form than in the bulk continuous phase because of the concentration gradient, due to consecutive adsorption of emulsifier onto the newly created (expanding) oil-water interface. The effective SC concentration at the MC outlet is less than 1/10 of bulk-phase concentration, as estimated according to the literature [38]: the effective concentration of SC in the emulsification part might be less than 0.1 wt%, even though the SC concentration in the bulk continuous phase is 1 wt%. Therefore, unstable emulsification at low SC concentrations is due to insufficient adsorption of SC molecules at the oil-water interface during droplet formation at the end of the MC.

#### 2.1.3. Effect of Emulsification Temperature

**Figure 4:**
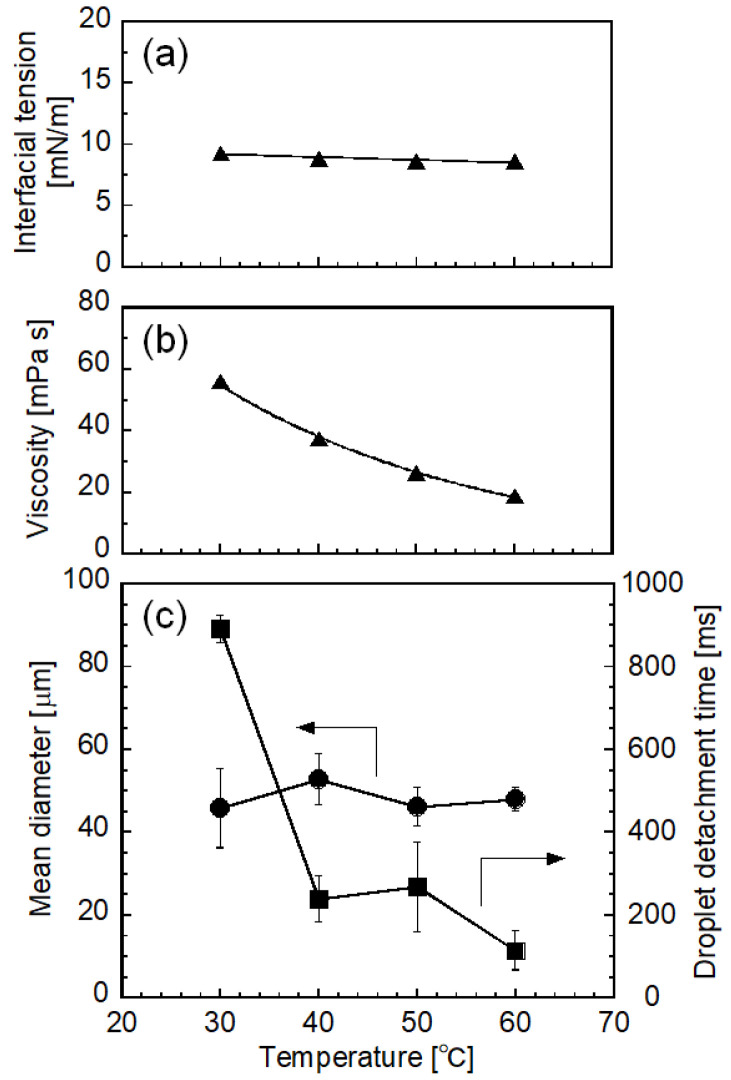
Effects of emulsification temperature on (**a**) interfacial tension (triangles), (**b**) viscosity of to-be-dispersed palm oil (triangles), and (**c**) *d*_m_ (circles) and droplet detachment time (squares) in the MC emulsification with MC-A using 3 wt% SC as an emulsifier. Error bars (**c**) denote standard deviations.

**Figure 5:**
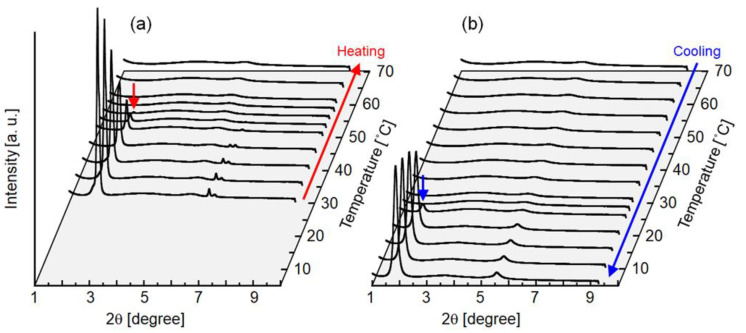
Small angle X-ray diffraction (SAXD) patterns of bulk palm oil used in this study recorded during (**a**) heating from 30 to 70 °C, and (**b**) cooling from 70 to 5 °C at a temperature variation rate of 2.5 °C/min. The short arrow on the peak at 2*θ* = 1.9° in (**a**) indicates the disappearance of a lamellar crystalline peak, and that in (**b**) indicates the appearance of a lamellar crystalline peak (lamellar length = 42 Å).

Figure 4 illustrates the effect of temperature on interfacial tension between the to-be-dispersed and continuous phases (Figure 4a), the viscosity of palm oil (Figure 4b), and the *d*_m_ and droplet detachment time (Figure 4c). Interfacial tension was not significantly affected by temperature in the examined range (Figure 4a). The viscosity of melted palm oil in bulk state decreased with increasing temperature. *d*_m_ varied from 45.7 to 52.8 µm; however, their temperature dependency was not obvious under the examined conditions. Emulsification was stable, and highly uniform oil droplets were generated at the end of the emulsification above 40 °C. Droplet detachment time [38,39] decreased with increasing emulsification temperature. At 30 °C, quite slow movement of to-be-dispersed palm oil on the terraces of MCs was observed; thus, droplet detachment required more time than at higher temperatures. In addition, monodispersity of the droplet size distribution decreased (CV > 20%) because some MCs produced large droplets. This emulsification behavior at 30 °C might be attributed to the partial crystallization of palm oil. As measured by small-angle X-ray diffraction (SAXD) (Figure 5), bulk palm oil exhibited crystallization between 25 and 27.5 °C under cooling, with the appearance of a sharp peak at 2*θ* = 1.9°, corresponding to the double-chain lamellar length (*d* = 42 Å, see Section 3.6 for details) of crystallized triglycerides in crude palm oil [22]. Even though the temperature of the emulsification module was maintained at 30 °C by circulating water, the actual temperature of the emulsification part would have a slight temperature distribution, which might cause partial crystallization of palm oil. Furthermore, during droplet formation in MC emulsification, palm oil flowed through MCs and transformed from a disk-like shape to a spherical droplet at the end of terraces. Under such conditions, shear flow in the narrow gap between the solid walls might accelerate solidification of the to-be-dispersed oil phase, as indicated by previous reports [13,40,41]. Emulsifier adsorbed on the oil-water interface might also affect crystallization of the oil phase [15,42]. Nevertheless, further research on the solidification of palm oil during emulsification at 30 °C is required for clarification.

#### 2.1.4. Effect of MC Structure

**Figure 6:**
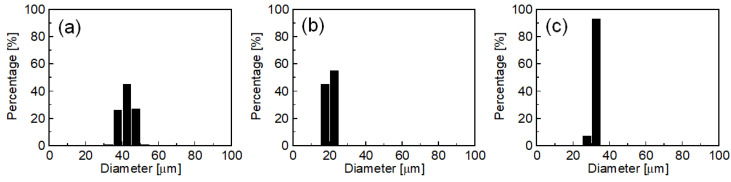
Number-weighted droplet diameter distributions of O/W emulsions obtained by MC emulsification using (**a**) MC-A, (**b**) MC plate type B (MC-B), and (**c**) MC plate type C (MC-C) (see Section 3.2). SC concentration was 3 wt%, and emulsification temperature was 60 °C. Each histogram contains data on diameter for more than 100 droplets.

Figure 6 presents the results of MC emulsification using different MCs (MC-A, MC-B, and MC-C, see “Materials and Methods” for details). These MCs had different geometries, which affected droplet diameter in MC emulsification [43,44]. Here, water containing 3 wt% SC and 0.2 M NaCl was used as the continuous phase (addition of 0.2 M NaCl affected neither droplet diameter nor interfacial tension). Successful emulsification producing uniform oil droplets was observed using all types of MC. The *d*_m_ was 42.6 µm (CV = 8.6%) for MC-A, 20.7 µm (CV = 6.3%) for MC-B, and 32.0 µm (CV = 3.5%) for MC-C. These results suggest that the droplet diameter in O/W emulsions can be controlled by using appropriate dimensions of MC.

#### 2.1.5. Effect of Flow Rate of To-Be-Dispersed Phase

**Figure 7:**
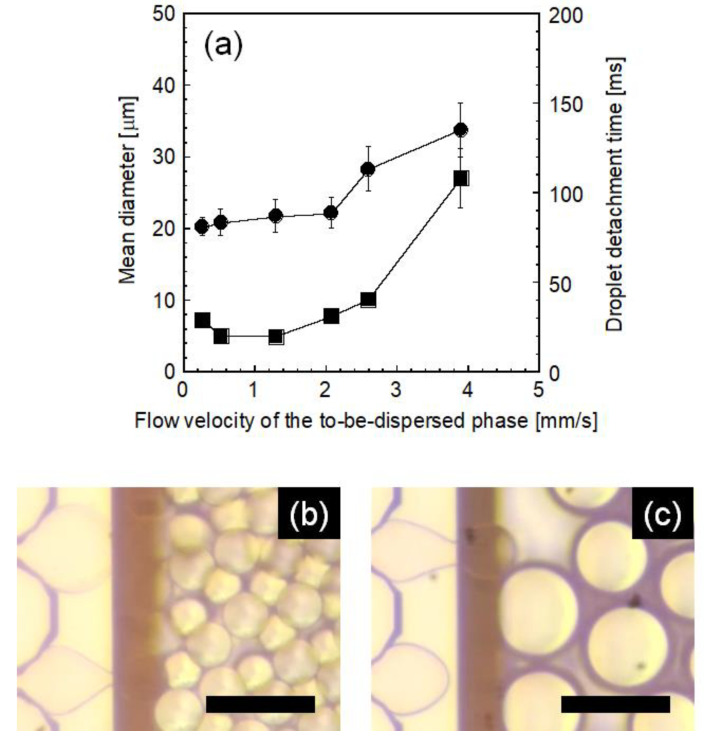
(**a**) Variation of mean diameter (*d*_m_, circles) and droplet detachment time (squares) as a function of the flow velocity of the dispersed phase that flows in a channel, and typical optical photomicrographs of droplets generated from channels (**b**) below the critical flow velocity (1.3 mm/s) and (**c**) above the critical flow velocity (3.9 mm/s). MC-B was used. SC concentration was 3 wt%, and emulsification temperature was 60 °C. The scale bars in (**b**) and (**c**) are 50 µm.

Here, the effect of the flow velocity of the dispersed phase that flowed through MCs was investigated to clarify the dynamic characteristics of droplet formation. Figure 7 illustrates the effect of flow velocity on the resultant droplet diameter and detachment time using MC-B. Monodisperse oil droplets were obtained, and a slight change in *d*_m_ was observed up to 2.1 mm/s, while *d*_m_ remarkably increased and droplet size uniformity decreased above this critical flow velocity. The droplet detachment time also increased remarkably above the critical flow velocity: detachment time at a flow velocity of 3.9 mm/s was five or more times higher than that at 1.3 mm/s, indicating that droplet generation differed greatly between these two conditions with flow velocities below and above the critical value. As indicated by photomicrographs, uniform and stable droplet generation was observed below the critical flow velocity (Figure 7b), whereas non-uniform droplet generation was observed above the critical flow velocity (Figure 7c). These results agree with those from previous studies [35,36,45]. As reported, critical flow velocity was analyzed in relation to the capillary number [45], which is defined as the relative magnitude of viscus force to interfacial force, although emulsification was affected by the physical properties of MCs [35] and the type of emulsifier [36]. In our study, the critical capillary number above which emulsification became unstable was determined to be 0.006. Based on this result, we conducted MC emulsification using a straight-through MC-C, and successful emulsification with high droplet productivity could be carried out at the flow velocity giving a capillary number smaller than the critical value.

### 2.2. Preparation of W/O/W Emulsions Containing Palm Oil

As the next step, we prepared a water-in-oil-in-water (W/O/W) multiple emulsion encapsulating fine water droplets containing a hydrophilic compound inside palm oil droplets. We conducted two-step emulsification using an ultrasonic homogenizer for primary W/O emulsification and an MC array device with MC-A for secondary (multiple) emulsification. Here, 0.2 M NaCl was added to both internal and external water phases to equilibrate the osmotic pressure between both phases.

**Figure 8:**
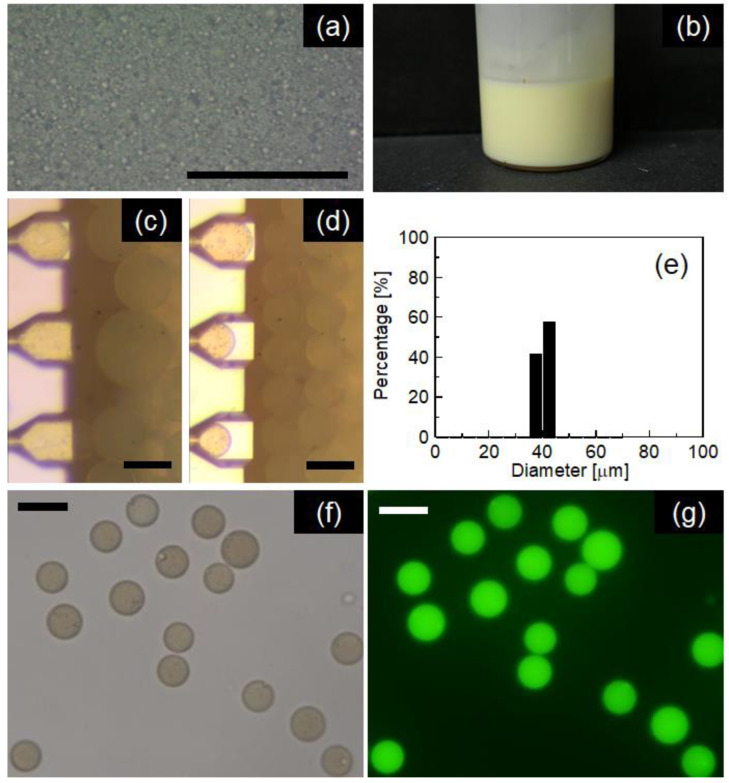
(**a**) Photomicrograph and (**b**) appearance of the primary W/O emulsion. Photomicrographs of emulsification for preparing W/O/W emulsions using MC-A at 60 °C with (**c**) 0.5 and (**d**) 3.0 wt% SC concentrations; (**e**) number-weighted droplet diameter distribution of W/O/W emulsion prepared under the same condition as (**d**); photomicrographs of W/O/W emulsions prepared under the same conditions as (**d**) and recovered from the MC emulsification module taken in (**f**) bright-field and (**g**) fluorescent modes.

The primary emulsification for preparing W/O emulsion was carried out using a 0.2 M NaCl aqueous solution containing 0.4 mM calcein as the dispersed phase and melted palm oil containing 3 wt% polyglycerin polycondensed ricinoleic acid ester (PGPR) as the continuous phase. The typical W/O emulsion presented in Figure 8a,b had a *d*_m_ of 1.36 µm with a polydispersity index of 0.21 determined by DLS measurement. Figure 8c,d presents the results of the preparation of W/O/W emulsions by MC emulsification using MC-C. The primary W/O emulsion was used as the dispersed phase, and 0.2 M NaCl aqueous solutions containing 0.5 or 3 wt% SC were used as the continuous phase. At 0.5 wt% SC concentration, multiple droplets were generated; however, their diameters were not uniform. In contrast, no droplet was obtained due to the continuous flowing out of the oil phase at the same SC concentration for O/W emulsification (Figure 3b). This difference in emulsification behavior might be attributed to the presence of PGPR in the oil phase of the primary W/O emulsion stabilizing the oil-water interface. At 3 wt% SC concentration, monodisperse multiple droplets with a *d*_m_ of 43.2 µm and a CV of 8.3% could be obtained (Figure 8d,e). Under a fluorescent microscope, the multiple droplets recovered from the emulsification module emitted green fluorescence derived from calcein dissolved in the internal water droplets (Figure 8f,g). The entrapment yield of calcein in the multiple droplets stabilized by 3 wt% SC was determined to be 85.0% with 3.17% as the standard deviation just after preparation. This high entrapment efficiency was attributed to low-shear, non-mechanical droplet formation in MC emulsification, as presented in previous reports including W/O/W emulsification with liquid oils [46].

## 3. Materials and Methods

### 3.1. Chemicals

Palm oil (refined, analytical standard grade), β-lactoglobulin (β-LG) from bovine milk (≧85%, lyophilized powder), and calcein were purchased from Sigma-Aldrich (St. Louis, MO, USA). SC (12.6–15.8% as N after drying), polyoxyethylene (20) sorbitan monooleate (Tween 80), SDS (>95%), and sodium chloride (NaCl) were purchased from FUJIFILM Wako Pure Chemical Corporation (Osaka, Japan). Hexaglycerin monolaurate (HGML) and polyglycerin polycondensed ricinoleic acid ester (PGPR) were kindly donated by Nikko Chemicals Co., Ltd. (Tokyo, Japan). Skimmed milk powder containing 31.6 wt% casein protein and 5.3 wt% whey protein (as solid fraction) was obtained from Megmilk Snow Brand, Co., Ltd. (Tokyo, Japan). The water used in all experiments was prepared using a Direct-Q water purifier system (Merck Millipore Corporation, Billerica, MA, USA) and had 18.2 MΩ cm resistivity. All other chemicals were purchased from FUJIFILM Wako Pure Chemical Corporation and were of extra-pure grade. All chemicals were used as they were obtained without further purification.

### 3.2. Preparation of O/W Emulsions

**Figure 9:**
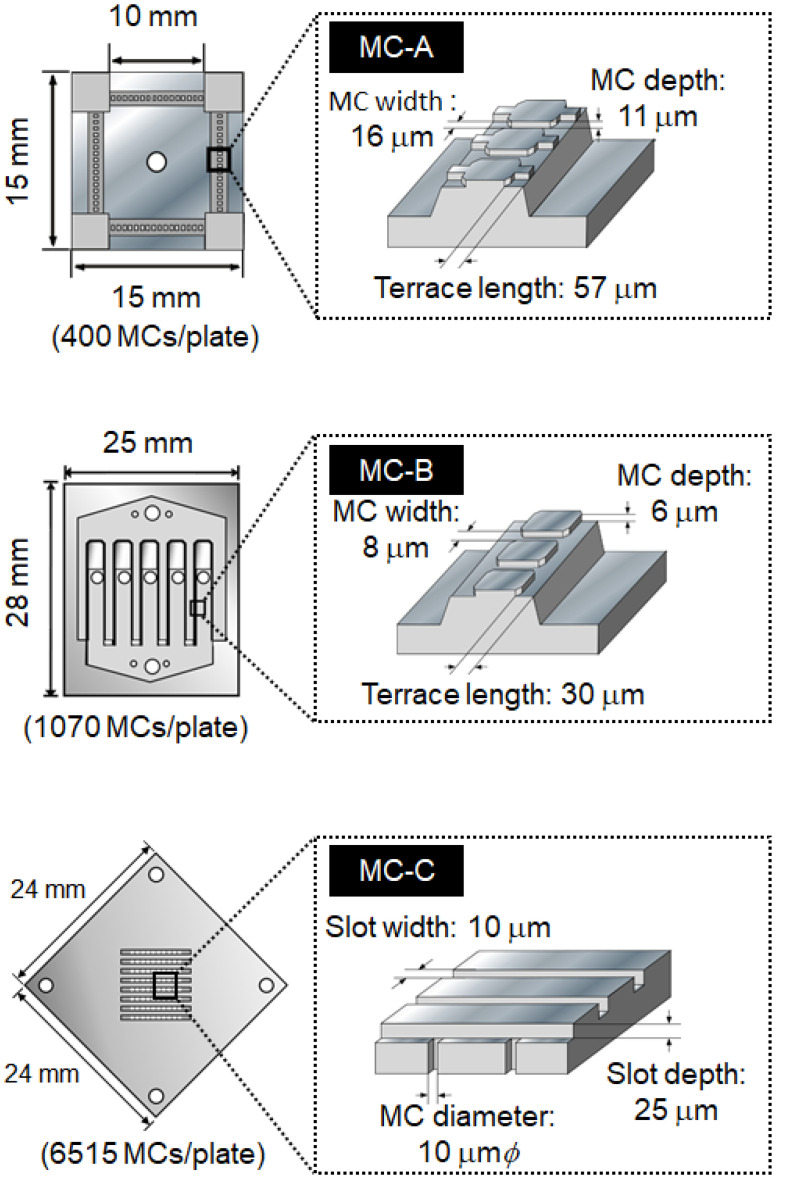
Schematic illustration and dimensions of MC plates used in this study.

O/W emulsions were prepared by MC emulsification using a laboratory-scale MC emulsification setup with grooved [24,25] or asymmetric straight-through [46] silicon MC plates (Figure 9). Two different grooved MC plates (dead-end type: MC-A, and cross-flow type: MC-B) were used in this study. The dimensions of the MCs (MC depth, MC width, and terrace length for grooved MC [44]; MC diameter, slit width, and slit depth for asymmetric straight-through MC [44]) fabricated on each silicon plate are also illustrated in Figure 9. The MC emulsification setup and silicon MC plates were purchased from EP-Tech (Hitachi, Japan).

Palm oil was preheated at 80 °C using a hot plate with magnetic stirring to liquefy it. The liquid oil was then loaded into a plastic syringe installed on a syringe pump and used as the to-be-dispersed phase. Aqueous solutions containing NaCl and emulsifiers were loaded into a glass syringe and used as the continuous phase. The emulsification module with an installed silicon MC plate and glass plate was initially filled with the continuous phase. Both oil and aqueous solutions were fed to the module using two syringe pumps (SPE-1, AS ONE Corporation, Tokyo, Japan). The plastic syringe containing palm oil and a plastic tube by which the syringe and the module were connected were heated to over 65 °C using a ribbon heater. The temperature of the emulsification module was controlled using a water-circulating constant-temperature jacket and monitored using a digital thermometer (AD 5624, A&D, Tokyo, Japan) during the emulsification experiments. The emulsification in the MC module was observed through a glass plate using a microscope video system equipped with a digital camera (Nikon 1 J1, Nikon, Tokyo, Japan) with a high-speed recording mode. Number-weighted *d*_m_ was determined using the measurement data obtained by measuring the diameters in the captured images of at least 100 droplets using Microsoft PowerPoint software. The CV was calculated based on the following equation:

CV [%] = l00 × σ/*d*_m_, (1)

where σ is the standard deviation of droplet diameter.

### 3.3. Preparation of W/O/W Emulsions

W/O/W emulsions were prepared using two-step emulsification: a combination of the primary ultrasonic emulsification and the secondary MC emulsification. To prepare the primary W/O emulsion, melted palm oil containing 3 wt% PGPR and an aqueous solution containing 0.2 M NaCl and 0.4 mM calcein were mixed in a glass bottle and homogenized for 10 min using a probe ultrasonic homogenizer (US-150, Nihonseiki Kaisha, Ltd., Tokyo, Japan). During homogenization, the sample bottle was heated to 80 °C on a hot plate with magnetic stirring. The droplet diameter of the primary W/O emulsion was measured using a dynamic light scattering particle size analyzer (ELSZ-1000, Otsuka Electronics Co., Ltd., Osaka, Japan). The secondary MC emulsification was carried out as described above, using the primary W/O emulsion as the to-be-dispersed phase and aqueous solutions containing 0.2 M NaCl and SC as the continuous phase. Prepared W/O/W emulsions were collected by flow of the continuous phase to a glass vial connected to the downstream outlet of the MC module. The entrapment yield of calcein into multiple droplets of W/O/W emulsions was determined by modifying a previous method [47,48]. The fluorescent intensity of calcein was determined using a spectrofluorometer (RF-5300PC, Shimadzu Corporation, Kyoto, Japan) as described previously.

The entrapment yield was calculated using the following equation:

(Entrapment yield) [%] = {(1.01 × *F*_inside_ − *F*_blank_)/(*F*_total_ − *F*_blank_)} × 100, (2)

where *F*_total_ is the fluorescence intensity of the W/O/W emulsion sample before adding CoCl_2_ solution (10 mM), and *F*_inside_ is that after adding the solution. Here, *F*_blank_ is the fluorescence intensity of the W/O/W emulsions themselves (i.e., those without calcein), which was measured separately. The dilution volume factor is 1.01, due to the addition of CoCl_2_ solution.

### 3.4. Viscosity Measurement

Viscosities of both the continuous phase and the dispersed phases were measured using a vibrational viscometer (SV-10, A&D, Tokyo, Japan). The temperature of the sample was controlled using a sample chamber with a water-circulating constant-temperature bath.

### 3.5. Interfacial Tension Measurement

Interfacial tension was measured at ambient temperature by the pendant drop method using an automatic interfacial tensiometer (DM-301, Kyowa Interface Science Co., Ltd., Niiza, Japan). The temperature of the sample was controlled using a stainless sample stage with a water-circulating constant-temperature bath. The time required to form the pendant just before detaching from the bottom edge of the needle was set to 5 s, in order to obtain reproducible values [39]. After the pendant drop formed, its image was captured, and interfacial tension was obtained using image analysis software FAMAS (Kyowa Interface Science Co., Ltd., Niiza, Japan). Each measurement was repeated at least 10 times, and the calculated mean values and standard deviations were used. Prior to measurement of interfacial tension, the density of each sample solution was determined using glass pycnometers in a temperature-controlled water bath.

### 3.6. SAXD Measurement

SAXD measurement using a synchrotron radiation source was carried out at beamline 6A at the Photon Factory of the High Energy Accelerator Research Organization (KEK, Tsukuba, Japan). The specifications of this equipment have been reported elsewhere [49,50]. The wavelength of the X-ray was 0.150 nm. X-ray scattering data were obtained using a PILATUS3 1M detector (DECTRIS Ltd., Baden, Switzerland).

Melted palm oil preheated at 80 °C for 30 min was placed between 1 mm-thick Kapton tapes, which were set to the sample stage of a thermocontrol system (Linkam Scientific Instruments Ltd., Surrey, UK). The distance between the sample and the detector was 900 mm. Analysis of the SAXD image data was carried out using SAngler software (KEK) [51,52]. Silver behenate was used as the standard specimen to calibrate the scattering angle. The length of the periodic structure in the sample, *d*, is calculated with the Bragg equation as follows:

*d* = *λ*/sin (2*θ*/2), (3)

where *λ* is the wavelength of the X-ray, and 2*θ* is the scattering angle.

## 4. Conclusions

In this study, we investigated the preparation characteristics of palm oil in water emulsion using various MC arrays under controlled temperature. Of the emulsifiers we examined, SC was the best one for obtaining monodisperse droplets. The dynamic behavior of droplet formation was affected by emulsifier concentration, temperature, and flow rate of the oil phase; however, three different MC geometries enabled us to obtain monodisperse oil droplets with a controllable droplet diameter. This approach could be extended to produce a W/O/W emulsion containing palm oil as the intermediate oil phase. The experiment details presented in this study demonstrate the factors affecting droplet generation in MC emulsification using palm oil, a temperature-responsive material. The results presented here will also be useful for producing size-controlled microspheres and microcapsules consisting of solid/semi-solid lipids, having high biocompatibility and controllable encapsulation/release properties derived from the melting behavior of palm oil under moderate temperature. Our findings will contribute to the manufacturing field in the food, pharmaceutical, and cosmetic industries.
